# The association between 2D:4D digit ratio and sex-typed play in children with and without siblings

**DOI:** 10.1038/s41598-024-65739-1

**Published:** 2024-07-02

**Authors:** Luisa Ernsten, Lisa M. Körner, Martin Heil, Nora K. Schaal

**Affiliations:** 1 Faculty of Mathematics and Natural Sciences, Institute of Experimental Psychology, Heinrich Heine University, Düsseldorf, Germany; 2 Department of General Pediatrics, Neonatology and Pediatric Cardiology, University Children’s Hospital, Medical Faculty and University Hospital Düsseldorf, Heinrich Heine University, Düsseldorf, Germany

**Keywords:** Psychology, Human behaviour

## Abstract

The 2D:4D digit ratio is commonly used as a surrogate possibly reflecting prenatal testosterone levels. Indirect evidence comes from studies investigating the association between 2D:4D and human characteristics that likely relate to prenatal testosterone. In children, sex-typed play reveals large sex differences early in development and an influence of prenatal testosterone is likely. Findings on the association between 2D:4D and children’s sex-typed play are heterogeneous and other influences on the development of sex-typed play have been suggested, most of all social influences like siblings, their sex and birth order. The current study examined the association between right and left 2D:4D, a proposed surrogate for prenatal testosterone exposure, which was assessed in right and left hands of *N* = 505 6-month-old children, and sex-typed play behavior, which was evaluated 3.5 years later using the Pre-School Activities Inventory (PSAI), and the influence of siblings. To capture differential effects of siblings’ sex and birth order, dummy-coded variables were used reflecting having no siblings as well as older or younger sisters or brothers. Multiple regression models were used to investigate the association between PSAI scores and sex, right and left 2D:4D, being a singleton as well as having an older or younger sister or brother. It was shown that sex and having an older brother were significant predictors for sex-typed play. Effects were further disentangled by conducting separate regression analyses in boys and girls. In boys, a significant association between PSAI scores and having an older brother was revealed, in girls, no significant associations were found. Results are discussed highlighting the non-significant association between 2D:4D and children’s sex-typed play, which weakens the applicability of 2D:4D as a surrogate reflecting influences of prenatal T. Further, the importance of social factors like siblings on children’s sex-typed play is discussed.

## Introduction

The 2D:4D digit ratio was introduced by Manning et al.^1^ who found a significant sex difference (males < females) and a negative association of 2D:4D with sperm count. The authors suggested that 2D:4D is likely modified by prenatal intrauterine testosterone levels, relatively stable across human development, and therefore a suitable marker for prenatal testosterone exposure^1^. Since then, the sexually differentiated pattern was replicated in various studies, however, with only moderate effect sizes^2^. As approaches for more direct evidences on the association between 2D:4D and prenatal testosterone are limited due to ethical considerations in humans, indirect approaches have been conducted in clinical samples. Studies suggested a masculinized (i.e., smaller) 2D:4D in individuals with altered intrauterine testosterone concentrations like individuals with congenital adrenal hyperplasia (CAH) who are exposed to high levels of testosterone during gestation^3–6^. Likewise, feminized (i.e., larger) 2D:4D was found in individuals with complete androgen insensitivity syndrome (CAIS)^7,8^, a clinical condition characterized by non-functioning androgen receptors. In these studies, effect sizes were generally small to medium and the variability of 2D:4D in clinical samples was comparable to healthy samples^3–8^. Other studies have focused on establishing associations between 2D:4D and other human characteristics as it is commonly assumed that androgens, especially testosterone, have organizational effects and shape brain connections already in-utero^9^.

Different studies have found significant associations between 2D:4D and aggression (see, e.g.,^10–12^, but see^13^), or athletic performances^14^ in adults. During childhood, sex-typed play behavior gathered special interest in past research. It shows considerably large effect sizes^15^, emerges as early as 1 year of age^16^, and a biological origin seems likely as it is also observable in non-human primates^17,18^. In human studies, it was shown that females with CAH show masculinized play behavior compared to unaffected females^19,20^. Further, individuals with CAIS show feminized behavior compared to non-affected controls^20^. Isolated gonadotropin-releasing-hormone deficiency (IGD), a condition with comparably low production of gonadal hormones during pregnancy, analogously was found to relate to less gender-conformity during childhood reported by affected adult males^21^. It is assumed that hormones like testosterone influence brain development as early as the fetal stage in such a way that higher levels of testosterone result in a masculinization, which can also affect behavior later in life^9^. Several studies investigated the direct association between sex-typed play and testosterone. Considering prenatal testosterone, different studies could not find a significant association of amniotic fluid testosterone levels with sex-typed play in toddlers^22^ and pre-school children^23,24^. Otherwise, Auyeung et al.^25^ found a significant positive relationship between maternal amniotic fluid testosterone concentrations and masculinized play behavior in on average 8.5-year-old boys and girls. Hines et al.^26^ obtained testosterone in maternal blood samples during pregnancy and found significant positive associations with male-typed play behavior at age 3.5 years, however, only in girls. Other studies investigated postnatal testosterone levels. Alexander and Saenz^27^ could not find a significant association between children’s salivary testosterone and different aspects of children’s play at age 3 to 4 months. Otherwise, Lamminmäki et al.^28^ found a significant association between urinary testosterone that was obtained weekly in newborn infants until 6 months of age and sex-typed play behavior at 14 months of age in the hypothesized direction (i.e., higher urinary testosterone related to more masculinized play behavior). Both studies have assessed testosterone during a time-frame where male testosterone levels peak while female testosterone levels remain low shortly after birth and possibly also have organizational effects on the brain (a time-frame also called 'mini-puberty’^29^). In sum, study results promote the idea that (prenatal) testosterone relates to sex-typed behavior during childhood even when measured with different techniques and approaches.

In children, different studies have examined the relationship between 2D:4D and sex-typed play. Some studies found that smaller 2D:4D was associated with more masculinized play behavior, however, only in boys^30,31^ or only in girls^32^, whereas other studies found it in both sexes^33^. Another recent study found no significant association between 2D:4D and sex-typed play behavior^34^. The studies used the same tool for the assessment of sex-typed play behavior, the elaborated Pre-School Activities Inventory^35^ (although Hönekopp and Thierfelder^30^ used a different method for the calculation of the total PSAI score). Although results should be highly comparable due to the methodology, results on the association between 2D:4D and children’s sex-typed play behavior remained heterogeneous.

With regard to differing study results, researchers suggested that controlling for other variables, like social influences, may influence the association between 2D:4D and sex-typed play behavior. Different models exist for the explanation of sex-typed behavior due to social influences (see, e.g.,^36^). Parents potentially have a crucial function as role models in children’s development and may also directly affect children by reinforcing or punish certain behavior^37^. While there is some evidence that mothers and fathers treat their children differently, Morawska^38^ found only limited evidence in their systematic review that children show different behavior due to gendered parenting. Further, social learning theories and social modelling, e.g., through siblings and peers, have been suggested to be important sources for the development of sex-typed behavior^39,40^. Research has found that siblings’ sex and birth order may be two relevant factors that affect the sibling relationship^41^. Stoneman et al.^42^ described differential behavior patterns of older and younger sisters and brothers in different sibling constellations. They found that same-sex siblings engaged in more gender-typical play, whereas other-sex siblings engaged in sex-typed play that was typical for the older sibling’s sex^42^. Similar results have been reported by Rust et al.^43^, who additionally investigated sex-typed play in singleton children. It was found that children with older same-sex siblings exhibited highest ratings of sex-congruent play behavior, whereas children with older opposite-sex siblings exhibited lowest ratings of sex-congruent play behavior^43^. Singleton children showed more sex-typed play behavior compared to children with opposite-sex siblings, however, less compared to children with same-sex siblings^43^. Less is known about the influence of younger siblings on older siblings’ sex-typed play behavior. Hughes et al.^44^ argued that younger siblings facilitate social skills in children, whereas Sang and Nelson^45^ found differential effects of younger siblings on older siblings and emphasized the importance to distinguish between different sibling constellations.

Regarding the association between 2D:4D and sex-typed play, some of the aforementioned studies also investigated the number and sex of siblings as an easy to quantify measure for potential social influences. Hönekopp and Thierfelder^30^ found no differences in sex-typed play behavior between children with brothers or sister. Mitsui et al.^31^ found that the existence of brothers or sisters was associated with more masculinized or feminized play behavior, respectively. Lastly, Körner et al.^32^ found that both 2D:4D and the existence of older siblings independently explained variance in sex-typed play, underscoring the importance to include both biological and social factors while investigating possible sources for sex-typed behavior. As the number and sex of siblings could be important for sex-typed behavior and is easily assessible, it seems reasonable to include this factor.

In sum, there is evidence that smaller 2D:4D, which presumably reflects more intrauterine testosterone exposure, can be associated with more masculinized play behavior in children, although, evidence differs substantially in the available literature. Further, it may be beneficial to investigate the influence of socializing factors, e.g., siblings, on sex-typed play. The aim of the current study was to investigate the association between 2D:4D and children’s sex-typed play. As past research found diverging results, the potential influence of siblings in the context of socialization and its effect on sex-typed play will be additionally investigated. A significant association between 2D:4D and children’s sex-typed play could be interpreted as a form of external validity for the applicability of 2D:4D as a marker for prenatal testosterone. However, as prior results substantially differ, it may be beneficious to include other variables that could affect this influence. Therefore, siblings are included as an additional factor. To capture diverging effects of siblings, different constellations of sibling’s sex and birth order will be incorporated. It is assumed in accordance to prior research that, specifically, older same-sex siblings relate to sex-congruent play behavior, whereas other-sex siblings possibly relate to sex-incongruent play behavior. As the influence of younger siblings has not been reviewed to a greater extent, this will be investigated in a more explorative way. Exceeding approaches of prior studies, the current study will also investigate the influence of being a singleton child on sex-typed play behavior, as research suggests that singleton children exhibit less sex-typed play behavior compared to children with same-sex siblings, however, more sex-typed play behavior compared to children with other-sex siblings.

## Methods

### Participants

As part of a larger study, German-speaking families with newborn children were recruited between 2013 and 2018 when infants were 6 months of age. Families were invited to the Institute of Experimental Psychology at Heinrich Heine University Düsseldorf, Germany, in order to take part in a mental rotation experiment (a subsample was published elsewhere, see^46^). In a follow-up 3.5 years later, parents were asked to rate their child’s sex-typed behavior with the PSAI using an online questionnaire, whether children have siblings and whether and for how long they attended pre-school. Children were on average 6 months old (*M* = 195.21 days, *SD* = 8.24) when hand scans were taken and on average 3.5 to 4 years (*M* = 46.25 months, *SD* = 3.74) when their parents completed the online questionnaire. All of the children except for *n* = 7 attended pre-school by the time of the online questionnaire. The online questionnaire was completed by mothers in 89.33% of the cases, by fathers in 5.33% and by both parents in 5.33% of the cases. Almost all families and infants were White and from middle-class backgrounds. All parents and/or legal guardians gave their written informed consent for participation. The current study was approved by the local ethics committee of the Faculty of Mathematics and Natural Sciences of Heinrich Heine University Düsseldorf, Germany, and was in accordance with the Declaration of Helsinki.

Hand scans were taken from a total of 1391 children (705 boys and 683 girls), which were also used for another work of our group^47^. For the current study, only complete data sets with valid hand measures of second and fourth digits of right and left hands as well as a completed online questionnaire were used, leaving *n* = 519 cases. Additionally, *n* = 5 cases of right 2D:4D, *n* = 4 cases of left 2D:4D, and *n* = 5 cases of the PSAI score were excluded as the score ranged more than +/- 2 IQR from the median, leaving a final data set of *n* = 505 (240 girls, 265 boys). Of these, *n* = 134 (66 girls, 68 boys) were singletons.

## Materials

### Digit ratio (2D:4D)

Both right and left hands of 6-months-old children were scanned by an examiner using a FUJITSU fi-60F image scanner. The ventral surface of right and left hands was independently pushed softly onto the scanner glass and covered with a towel to increase contrast. For determining the length of digits, the freeware program *AutoMetric*^48^ was used. For each digit, the length between the midpoint of the ventral proximal crease to the tip was measured. A 100 dpi monitor was used (100 pixels = 2.54 cm). The 2D:4D digit ratio was estimated by dividing the length of the second digit (2D) by the length of the fourth digit (4D). Indirect measurement techniques like hand scans can achieve larger sex differences and higher measurement precision than direct measurements^49^. Further, *Autometric* shows a high reliability for digit measurements and is superior to other computer-based measurement techniques^32^. Hand scans were rated by two independent raters, who were blind to the sex of the children. Intra-class correlations varied between 0.95 and 0.97, which can be seen as almost perfect. The two ratings were averaged for each digit on each hand to increase reliability.

### Preschool activities inventory

The Preschool Activities Inventory (PSAI) is a standardized questionnaire by Golombok and Rust^35^ used to measure sex-typed play behavior in children. In the present study, the German version described by Hönekopp and Thierfelder^30^ was used. Sex-typed play behavior is measured via 24 items, divided into three groups: 7 toy items (e.g., ‘guns’, ‘jewellery’), 11 activity items (e.g., ‘fighting’, ‘playing house (e.g., cleaning, cooking)’) and 6 character items (e.g., ‘likes to explore new surroundings’, ‘likes pretty things’). Twelve items each are characterized as typically male and female. Parents were asked to rate the preferences of their child for the different toys and actions and their child’s temperamental characteristics on a 5-point Likert-scale. A higher score indicates more typically male play behavior, a lower score more typically female play behavior^35^. The total PSAI score was calculated as follows according to Golombok and Rust^35^:

1 $$PSAI score = 48.25 + 1.1 (sum of "\text{male" }items - sum of \text{"female" }items)$$

### Procedure

After arrival, written informed consent of caregivers was obtained. Next, the mental rotation task was performed^46^. Examiners then took hand scans of both right and left hands from children without giving further information about the interpretation of their childrens’ finger length. Parents received a refund of their travel expenses. Families were contacted again approximately 3.5 years later. Parents followed an invitation via e-mail and answered a questionnaire using an open source online survey tool^50^ and, again, gave their informed consent for participation, recording, and storage of data. Then, parents were asked to answer an online questionnaire including the PSAI^35^. Further, they stated who filled out the questionnaire, whether and for how long the child attended preschool, and whether and how many older and/or younger brothers and/or sisters the child had. The study was approved by the local Ethics Committee of the Faculty of Mathematics and Natural Sciences of the Heinrich Heine University Düsseldorf, Germany.

### Statistical analyses

To test whether 2D:4D and PSAI scores differed between the sexes, independent samples *t* tests were conducted.

To test for the influence of siblings on sex-typed play behavior, a multiple linear regression model with the criterion PSAI score and the predictors *sex*, *2D:4D* (right and left), *singletons* (dummy coded as 0 = no singleton, 1 = singleton), *older sisters* (dummy coded as 0 = no older sisters, 1 = at least one older sister), *younger sisters* (dummy coded as 0 = no younger sisters, 1 = at least one younger sister), *older brothers* (dummy coded as 0 = no older brothers, 1 = at least one older brother), and *younger brothers* (dummy coded as 0 = no younger brothers, 1 = at least one younger brother) was tested. Pearson correlations are reported for all study variables (PSAI score, sex, right and left 2D:4D, and dummy-coded variables singletons, older sisters, younger sisters, older brothers, younger brothers). To further disentangle effects of sex, two separate multiple linear regression models incorporating the same predictor variables (right and left 2D:4D, singletons, older sisters, younger sisters, older brothers, younger brothers) were run separately for subsamples of boys and girls.

Levels of significance were set to 0.05 for all comparisons. Pearson’s *r* was interpreted as follows: small effect *r* ≥ 0.10, medium effect *r* ≥ 0.30, and large effect *r* ≥ 0.50^51^. Cohen’s *d* was interpreted as small effect *d* ≥ 0.20, medium effect* d* ≥ 0.50, and large effect *d* ≥ 0.80^51^.

## Results

Right and left 2D:4D as well as PSAI scores were tested for normal distribution in boys and girls separately using Shapiro–Wilk tests and revealed no violation for normality assumption (all *p* ≥ 0.173).

First, sex differences in 2D:4D and PSAI scores were tested for significance. Overall, girls had larger digit ratios and lower PSAI scores compared to boys. There were no significant sex differences in the overall sample in left 2D:4D, *t* (503) = 0.92, *p* = 0.180, *d* = 0.08, only right 2D:4D differed significantly between the sexes, *t* (503) = 1.67, *p* = 0.048, *d* = 0.16. PSAI scores differed significantly between girls and boys of the overall sample, *t* (503) = 29.16, *p* < 0.001, *d* = 2.60.

The association between sex-typed play behavior and siblings was further analyzed. Chi-square tests revealed that boys and girls did not significantly differ in the number of older and/or younger sisters and brothers (all *p* > 0.173, two-sided). Pearson correlations of the study variables are shown in Table 1. The multiple linear regression model with the predictors *sex*, *right 2D:4D*, *left 2D:4D*, *singletons*, *older sisters*, *younger sisters*, *older brothers*, and *younger brothers* was overall significant, *F* (8, 496) = 112.03, *p* < 0.001, with an *R*^*2*^ = 0.64 (adjusted *R*^*2*^ = 0.64). PSAI scores could be significantly predicted by the factors *sex*, and *older brothers (*see Table 2 for the results of the multiple linear regression model)﻿.﻿ To detect differential influences of siblings on PSAI scores of boys and girls, the multiple regression analysis was again tested separately for boys and girls using the predictors *right 2D:4D*, *left 2D:4D*, *singletons*, *older sisters*, *younger sisters*, *older brothers*, and *younger brothers*. In boys, the overall model was significant, *F *(7, 257) = 3.54, *p *= .001, with an *R²* = .09 (adjusted *R²* = .06). For boys, only having older brothers was a significant predictor of the PSAI score (see Table 3). For girls, the overall model did not reach significance, *F *(7, 232) = 0.63, *p *= .734, with an *R² *= .02 (adjusted *R²* = −.01). None of the predictors reached statistical significance (see Table 3).

**Table 1:** Pearson correlations of study variables.

Variables				1	2	3	4	5	6	7	8
1	PSAI			–							
2	Sex			− 0.79***	−						
3	2D:4D		right	− 0.07	0.07*	-					
4			left	− 0.06	0.04	0.42***	–				
5	Singletons			0.01	0.02	0.11**	− 0.01	–			
6	Sisters		older	− 0.05	0.03	0.03	− 0.00	− 0.29***	–		
7			younger	− 0.08*	0.03	− 0.11**	− 0.04	− 0.30***	− 0.16***	–	
8	Brothers		older	0.10*	0.00	0.00	− 0.01	− 0.33***	0.00	− 0.16***	–
9			younger	0.00	− 0.04	− 0.03	0.05	− 0.32***	− 0.17***	− 0.25***	− 0.16***

*N* = 505; *** *p* < 0.001, ** *p* < 0.01, * *p* < 0.05 (two-tailed).

**Table 2:** Results of the multiple linear regression model testing the association between sex, 2D:4D, and siblings on PSAI scores.

Variable					95% CI		*t*	*p*
		*B*	*SE*	β	Lower	Upper		
Intercept		73.61	13.15		47.77	99.44	5.60	< 0.001***
Sex		− 25.91	0.88	− 0.79	− 27.64	− 24.18	− 29.37	< 0.001***
2D:4D	right	− 5.79	12.74	− 0.01	− 30.81	19.23	− 0.46	0.650
	left	− 7.48	13.14	− 0.02	− 33.30	18.33	− 0.57	0.569
Singletons		2.11	1.88	0.06	− 1.59	5.80	1.12	0.264
Sisters	older	− 0.59	1.57	− 0.01	− 3.67	2.49	− 0.38	0.707
	younger	− 0.99	1.76	− 0.02	− 4.45	2.47	− 0.56	0.576
Brothers	older	4.40	1.50	0.11	1.46	7.35	2.94	0.003**
	younger	− 0.04	1.73	− 0.00	− 3.44	3.37	− 0.02	0.983

The variables singletons (0 = not a singleton, 1 = singleton) as well as *older sisters*, *younger sisters*, *older brothers*, and *younger brothers* were entered as dummy-coded variables (0 = no older/younger sister/brother, 1 = at least one older/younger sister/brother).

*** *p* < 0.001, ** *p* < 0.01.

*N* = 505;

**Table 3:** Results of separate multiple linear regression models testing the association between 2D:4D and siblings on PSAI scores for boys and girls.

Model	Variable			β	95% CI		*t*	*p*
		*B*	*SE*		Lower	Upper		
Boys								
Intercept		79.13	17.16		45.35	112.92	4.61	< 0.001***
2D:4D	Right	− 13.09	16.72	− 0.05	− 46.03	19.84	− 0.78	0.434
	Left	− 4.51	17.55	− 0.02	− 39.08	30.05	− 0.26	0.797
Singletons		1.53	2.56	0.07	− 3.50	6.56	0.60	0.550
Sisters	Older	− 3.05	2.22	− 0.12	− 7.41	1.32	− 1.38	0.170
	Younger	− 3.55	2.40	− 0.15	− 8.27	1.18	− 1.48	0.140
Brothers	Older	4.45	2.00	0.20	0.52	8.38	2.23	0.027*
	Younger	− 1.71	2.37	− 0.08	− 6.38	2.96	− 0.72	0.472
Girls								
Intercept		43.79	20.36		3.66	83.91	2.15	0.033*
2D:4D	Right	− 1.07	19.70	− 0.00	− 39.88	37.74	− 0.05	0.957
	Left	− 9.05	19.87	− 0.03	− 48.19	30.09	− 0.46	0.649
Singletons		2.19	2.82	0.09	− 3.36	7.73	0.78	0.438
Sisters	Older	1.25	2.25	0.05	− 3.19	5.68	0.56	0.580
	Younger	0.91	2.62	0.04	− 4.26	6.08	0.35	0.728
Brothers	Older	4.03	2.30	0.17	− 0.50	8.56	1.75	0.081
	Younger	1.06	2.57	0.04	− 4.01	6.13	0.41	0.680

The variables singletons (0 = not a singleton, 1 = singleton) as well as *older sisters*, *younger sisters*, *older brothers*, and *younger brothers* were entered as dummy-coded variables (0 = no older/younger sister/brother, 1 = at least one older/younger sister/brother).

***p < 0.001, *p < 0.05.

*n* = 265 boys and *n* = 240 girls.

A sensitivity analysis revealed that, given an α error probability of 0.05, a power of 0.95, the total sample size *N* = 505 and a total of eight predictors in the analysis, an effect size *f*^*2*^ = 0.05 can be found with λ = 23.10 and a critical *F* (8, 496) = 1.96.

## Discussion

The current study aimed to examine the relationship between 2D:4D digit ratio measured at 6 months of age and concurrent sex-typed play behavior of nearly 4-year-old girls and boys, as well as the influence of siblings by investigating the influence of having no siblings, older sisters, younger sister, older brothers and younger brothers. In the overall sample, only small sex differences could be found in 2D:4D (males < females) and this difference was only significant in right hands. The sex difference in PSAI scores, however, could be considered large. As predicted, boys were reported to exhibit more masculinized play behavior, while girls were reported to show more feminized play behavior. The regression model in the overall sample was significant and revealed a significant influence of sex and that having an older brother related to significantly more male-typed play behavior in the current sample. The effect of older brothers on male-typed play behavior was also found in the subsample of boys, in girls, none of the predictors was significantly associated with PSAI scores.

Our analysis revealed only small or non-significant sex differences in 2D:4D, which contradicts the assumption of sex differences regardless of age and aligns with studies indicating small to non-existent sex differences in 2D:4D of very young cohorts^34,52,53^. Several studies have indicated that sex differences in 2D:4D become larger with age (see, e.g., Butovskaya et al.^54^, Ernsten et al.^55^, Trivers et al.^56^; but see also Lolli et al.^57^, and Forstmeier^58^, for the complexity of human allometry and how it can be reflected in simple ratios), therefore, it may not be surprising that we did not find sex differences in this young cohort of 6 months old children. It is commonly assumed that sex differences in 2D:4D are reliably detectable after the age of 2 years^1,59,60^, whereas in younger cohorts only small or non-existent sex differences can be obtained^52^. In younger cohorts, some authors already suggested that measurement techniques may contribute to difficulties in finding larger effect sizes, as a higher amount of soft tissue in very young children possibly impairs the reliability of obtained hand scans^61^. Furthermore, standardized measurements can be more difficult to perform in very young cohorts as adherence to instructions possibly lacks^47^. McIntyre et al.^62^ found the digit ratio of the third to the fourth finger (3D:4D) to be a significantly better discriminator between boys and girls and argue that the measurement of longer digit segments possibly produces less measurement error compared to shorter digits. In a prior study, McIntyre et al.^53^ found sex differences in both 2D:4D and 3D:4D, however, the sex difference in 3D:4D was evident much earlier compared to 2D:4D (i.e., 3D:4D at 5 years and 2D:4D at 9 years of age). Therefore, 2D:4D may not be the best marker to detect sex differences in very young cohorts. In sum, the cohort of the current study seemed to be in a critical age concerning the reliable detection of sex differences in 2D:4D. Nevertheless, although the current study did not find large sex differences, a descriptive difference could be found in the hypothesized direction (i.e., males < females) in our sample for both the right and left 2D:4D.

The sex difference in sex-typed play could be considered large, with significantly more feminized play behavior in nearly 4-year-old girls, and significantly more masculinized play behavior in nearly 4-year-old boys according to parents’ reports. This aligns with the broad body of literature^9,15,30–34^ and emphasizes the overall finding of large sex differences in sex-typed play behavior in pre-school children.

The question of interest of the current study, however, was the association between sex-typed play and 2D:4D as a surrogate of prenatal testosterone exposure and incorporating different sibling constellations as a possible social influence. 2D:4D and sex-typed play behavior did not relate in our sample. Also Barrett et al.^34^ did not find any significant association between 2D:4D and sex-typed play, whereas Wong and Hines^33^ found it in both sexes. However, both study groups did not investigate the influence of siblings. Hönekopp and Thierfelder^30^ as well as Mitsui et al.^31^ reported significant associations between 2D:4D and overall PSAI scores only in boys, with Hönekopp and Thierfelder^30^ reporting no significant associations between siblings and PSAI scores, while Mitsui et al.^31^ found that older same-sex siblings related to more sex-congruent play behavior in children. Both studies used correlational analyses between PSAI scores and sibling variables. Körner et al.^32^ reported a significant association between 2D:4D and sex-typed play as well as that older brothers significantly related to more male-typed play behavior, however, only in girls. These authors also used multiple regression models incorporating both 2D:4D and sibling variables as predictors for PSAI scores. Körner et al. ^32^ argued that effects of variations of prenatal testosterone in a normal range may be evident only up to a specific threshold^32^ and that in boys, who are exposed to generally higher levels of prenatal testosterone, this variation possibly has no further effect^26,32^. While our study results agree with the seemingly greater influence of older siblings, in the current sample, this was only true for having older brothers. Also Rust et al.^43^ described that older brothers may reveal a larger effect on sex-typed play compared to older sister. They outlined that these differential effects may stem from cultural variations in how gender-normative or non-normative behavior is accepted in girls and boys^43^. A comparable result was found by Braun and Davidson^63^, where boys who conformed to gender norms were most liked by their peers, whereas boys who did not conformed to gender norms were least liked. Conversely, girls who did not conform to gender norms were most liked by peers^63^. In sibling constellations, this may suggest that male siblings possibly act in a way that is more sex-congruent, whereas female siblings display a broad spectrum of behaviors and therefore become less sex-congruent.

In a more explorative approach, the current study included being a singleton and having younger siblings as additional variables that have not been investigated to a greater extent in other studies. However, these sibling constellations did not reveal any association with sex-typed play in children of the current sample. Rust et al.^43^ discussed the differential influence of siblings on sex-typed behavior and argued that same-sex siblings and specifically older siblings shape sex-congruent behavior while children without siblings seem to be more variable in their sex-typed behavior, however, show more sex-congruent behavior compared to children with siblings of the opposite sex. The existence of younger siblings, on the other hand, possibly encourages nurturing and more caregiving behavior regardless of sex^44^, a behavior that is typically characterized as feminine. It is further possible that children with younger siblings engage to a greater extent in sibling differentiation, which means to engage in sex-incongruent behavior to set oneself apart from the same-sex sibling^43^. Girls seemed to be less influenced in sex-typed play by siblings of either-sex in the current sample, and there are different studies on whether there is a difference in reinforcing or attenuating sex-typed behavior (e.g., through parents) between girls and boys (see, e.g.,^64,65^). A recent review, however, did not find robust evidence for differential reinforcement of sex-typed behavior between girls and boys^38^.

In the current study, the statistical effect of older brothers exceeded the effect of 2D:4D on sex-typed play behavior, with 2D:4D having no significant effect on sex-typed play behavior. One could argue that the effect of social influences, like siblings, has prevailed the effect of biological factors as indicated by 2D:4D as a surrogate for prenatal testosterone on sex-typed play behavior in the current study. This would underscore the importance of social influences on pre-school children’s sex-typed play behavior^66,67^. The effect size of 2D:4D was possibly too low to reliably differentiate between the sexes and to reveal a significant association with sex-typed play, especially when social influences like siblings were present. A larger sample could be indicated, although other studies found the hypothesized significant findings in a smaller sample compared to the current^32^.

The current study had some limitations. First, the study population was relatively homogenous in terms of ethnicity and socio-economic status. Specifically as 2D:4D significantly varies with respect to ethnicity^68^, results may not be generalizable. Next, the PSAI is a tool for the assessment of children’s sex-typed play by reports of parents, therefore, a distortion by parents’ perceptions of gender norms cannot be ruled out and, additionally, parents’ perception of their child’s play behavior may be influenced by siblings and subsequently by comparisons between children. Sex-typed play in children is a multifaceted construct and can be measured or observed in many ways. Other researches have used other tools to assess sex-typed play, like choices and preferences of playing tools^22,27^, and also found associations with 2D:4D^27^. Also the PSAI itself captures sex-typed play on different subscales (toy, activity, and character items, see^35^), which are used to calculate an overall score that should represent children’s sex-typed play. However, the PSAI possibly does not capture the multifaceted nature of sex-typed play or, more generally spoken, of gender role behavior of children. Therefore, other measures for sex-typed play or gender role behavior and their association with 2D:4D should be investigated in future studies. The current study did not account for other social or cognitive influences that may impact the development of sex-typed play, like other same-sex or other-sex peers, teachers, other family members, or sex-role identities of the children^69^, as well as for gender and/or sex diversity. The literature discusses non-binary and genderqueer sexual differentiation and identification that go beyond the scope of the current study. Nevertheless, sexual differentiation and identification other than the binary conception are important aspects to have in mind while discussing the source of sex/gender differentiation. A more general limitation could be the usefulness of 2D:4D as a marker for prenatal testosterone itself. Evidence in humans for direct associations between 2D:4D and prenatal testosterone is scarce and heterogeneous^55,70–72^, although, some evidence exists and 2D:4D is a commonly used surrogate to investigate associations of prenatal testosterone with other variables of interest^73^. As effect sizes between 2D:4D and sources of prenatal testosterone have to be generally considered as small^73^, there seems to be another source of variance that has not yet been identified. Also Hönekopp and Watson^2^ noted the significance of identifying these other sources that may affect 2D:4D in one of the first meta analyses on 2D:4D and to control for them in future studies. Therefore, the results have to be interpreted with caution.

In sum, the results of the current study suggest to critically review the usefulness of 2D:4D as a proxy for prenatal T, as no significant associations were found between 2D:4D and sex-typed play in the current study. Further, the results of significant influences of older brothers on more male-typed play behavior further promote the importance of social influences on children’s sex-typed behavior.

## Data Availability

The datasets analyzed in the current study are available in the Open Science Framework repository, https://osf.io/3cja7/.
